# New breast milk in old bottles

**DOI:** 10.1186/1746-4358-3-9

**Published:** 2008-08-04

**Authors:** Barbara Katz Rothman

**Affiliations:** 1 Department of Sociology, Baruch College, City University of New York, USA

## Abstract

This paper identifies how the different ideologies of patriarchy, technology, capitalism, race and feminism shape how we see breastfeeding and the breastfeeding mother with child. Ultimately, while we can make good strong arguments for breastfeeding from the perspective of health, of outcome, of good scientific data, we need to appreciate that they are only rationalizations for a shared belief that the image of the breastfeeding woman with baby represents something precious and valuable. So while it may be important to make arguments that draw on what is valued in society, we need to think hard about what it is that we value so that as we move forward with our efforts to make breastfeeding safe, we can use but not be used by, the various ideologies or claims.

## Introduction

Bear with me for a moment, and fill in the blanks below:

The rival brand to Pepsi is ______.

A little jab in the ribs is a _____.

A puff on a joint is a _____.

A short funny story with a punch line is a ______.

The white of an egg is the _______.

Just about everybody answers that the white of an egg is the yolk. And, just about everybody knows that is not true. The point of this exercise is to drive home the idea that what we see/hear/think is very context-dependent.

And now, another thought exercise: Picture a small room, with one comfortable chair in the center, and in that chair a woman with a baby at her breast. Please hold that image in your mind through everything I now say. That room has several doors leading into it. Which door you are coming in – "where you are coming from" – will shape what you see in that room. I am going to identify just a few of the many doors from which we may be entering, and consider how they shape what we see. Each of these doors represents a shared way of thinking, what we call "an ideology." Ideologies are political; that is, they rest on a power base. When people internalize ideologies, they become part of "common sense," what "everyone knows," and what needs no explanation. The doors, or ideologies, I am going to examine include patriarchy, technology, capitalism and race.

## Patriarchy

Patriarchy is one of the easiest to understand of the ideologies that shape motherhood and the understanding we bring when we open the door to that suckling woman. More than half the world has someplace to stand, another reality – women's reality – to contradict this particular ideology. But, women's reality is not the dominant ideology, and women's view of the world is overruled by men's view. Motherhood in a patriarchal society becomes what mothers and babies signify to men. The term "patriarchy" sounds almost quaint now, a relic of 1970s feminism. But in anthropological work, the term technically means "rule of fathers," not just rule of men. And, when the subject is mothering, there is an important difference. Patriarchal kinship systems rank paternity as the central social relationship: children are born to men, out of the bodies of women, and women, in this system, bear the children of men. In such a system, because what is valued is the relationship of men to their sons, women are a vulnerability that men have: to beget these sons, men must pass their seed through the body of women.

Perhaps even more important in shaping what we see when we open the door to a breastfeeding mother, in patriarchy it is the "seed" that is the essence, and nurturance is reduced to "soil," to "dirt," to meaningless background which can only take-away from, never add to, the intrinsic value of the grown being. In pregnancy, and in their motherhood, women can only ruin, hurt, destroy, damage their babies, or allow them to flower to the fullness of the essence they had from their seed. Mothering in patriarchy – and reflected so powerfully in medical care – is a risk, not an act of creation.

## Technology

One definition of technology is that it is just a tool, not good and not bad, but just a neutral tool that can be used for whatever purpose. Yet neutrality is not consistent with the other attribute we ascribe to technology: its practicality. Technology is the application of science, supposedly pure science, applied to practical ends. But as soon as one concedes that technology is *for *something, then it is no longer neutral. The ideology of a technological society is not just a package of tools, gimmicks, know-how; it is a way of thinking about the world in mechanical, industrial terms. The use of mechanical, industrial metaphors influences so many aspects of our lives: organizations working like "clockwork," people "programmed" to think in certain ways, bodily "plumbing." With changing times, the prototypes for the machines change, and along with it our fears and fantasies, from the runaway conveyor belt of a Charlie Chaplin film to HAL taking over in *2001: A Space Odyssey*.

And all this shapes how we see ourselves and our children. The world and all that it contains, including our own bodies, ourselves and our children, become potential resources, something to *make something of*. We build our bodies, sharpen our wits, and work on our relationships – and on our children. Efficiency is a crucial value in such a system, and we apply our ideas about machines to people, asking them too to be more efficient, productive, rational, and controlled. When a doctor manages a woman's labor, controlling her body with drugs and surgery, it is precisely to make her labor more efficient, predictable and rational. And so it follows that mothers push their babies onto a schedule – so that feeding the baby meshes into the "nine-to-five" day. We organize our time, our days, our lives and our relationships. We divide up tasks, and do them in the most efficient way possible: perhaps one woman expressing milk, another feeding it to the baby, in the interests of better "time management" for all.

## Capitalism

The ideology of technology dehumanizes people by encouraging a mechanical self-image: breasts, in this case, as machines for the production of milk – women's nipples and babies' lips as transport systems. Capitalism adds that not only is the body a collection of parts, but these parts become commodities. In the United States the essential fluids of life – blood, milk, and semen – are all for sale. There is a price tag on everything. Similarly, relationships become services, available for purchase or hire. Need arms to hold a baby, need someone to wield an alternative transport system for breast milk? Hire them. And, given the patriarchal focus on the seed, now expanded to include the highly valued seeds of some (educated, upper class) women, the nurturance work that is being hired is cheap and devalued.

Capitalism is an ideology as much as it is an economic system, and we see that in the deep ways it influences our thinking and language in such a wide variety of areas. "Choice," the language of the marketplace, is the only acceptable language to use to demand individual respect and autonomy. Second wave feminists saw that, and used it very effectively: feminism and self-determination for women became a "choice," and it is very difficult to argue against anyone's "choice" in a system ruled by this ideology. In such thinking, power is allowed to slip below our radar: people have to be truly free to "choose," and the power that would give them genuine freedom – and thus meaningful choices – is not discussed.

## Race

Race, and most assuredly racism, is itself an ideology, a way of thinking about and ordering the social world. The American history of race is something we bring with us as we open that door to a suckling woman. A 1993 Cultural Studies conference at the University of Michigan used an image, taken from an advertisement for "United Colors of Benetton," of a black woman suckling a white baby. The University's Women of Color Task Force objected. Patricia Williams was one of the people who responded to the controversy. What would it mean, she asked, if we were to reverse the image, placing a black baby at the white breast?

Is there not something unseemly, in our society, about the spectacle of a white woman mothering a black child? A white woman giving totally to a black child; a black child totally and demandingly dependent for everything, sustenance itself, from a white woman. The image of a white woman suckling a black child; the image of a black child suckling for its life from the bosom of a white woman. The utter interdependence of such an image, the merging it implies; the giving up of boundary; the encompassing of other within self; the unbounded generosity and interconnectedness of such an image[1].

As a white adoptive mother of a child of African American descent, I have spent many hours of my life staring at exactly that, my black baby suckling at my white breasts, and thinking about it. What am I looking at in that reverse image? Unbounded generosity? Interconnectedness? Some weird gendered version of white man's burden? Genocide? A better Benetton ad?

I cannot, still, after all these years and all this thinking, quite disentangle that image. But the image of the white baby at the black breast, now *that *is an image with a powerful American back story, the enslaved wet nurse, the woman whose own babies were allowed to hunger and even die, as they nursed their little white charges. That is the image that many of us hold in our heads as we look at a black or brown woman carefully defrosting the precious white milk of her employer and feeding it – not at breast but with bottle – to the white child. That image and that history shapes what we see when we see a suckling baby.

## Feminism

Feminism itself is an ideology, a way of thinking about the world, and represented an attempt – more or less successful – to grapple with each of these ideologies. Many would argue that it was most successful in dealing with patriarchy, and so simply moved more women into the position of fathers, exacerbating in many ways the problems brought to us by ideologies of capitalism, technology and most especially race.

But feminism must be seen as one of the doors we enter through. Many of us at the symposium are very much the product of the feminism of the 1970s, and some of us – such as Judy Norsigian representing *Our Bodies Ourselves *– are themselves the causes and not just the products of that feminism. That second wave feminism asked us to stand in all those places, more or less critically, and think about mothering, and about breastfeeding.

My oldest child was born in 1974. I was strongly committed to a personally-empowered home birth. But I very clearly remember reading "The Womanly Art of Breastfeeding" while I was pregnant, and thinking, "Well, I want to breastfeed anyway" – in spite of the good arguments of La Leche League. It is, frankly, much the way I feel about the current public health arguments: I want women to breastfeed anyway, however troubling the arguments and propaganda being offered.

## Conclusion: why care?

The woman in that room is threatened. Breastfeeding rates are low, and getting lower among some groups. If it is not the sanctity of motherhood and women's ultimate feminine roles that persuades me to try to keep her safe, and it is not the emphasis on "healthy babies," that persuades me, what is it then that I value about that image of a woman with baby at breast? We were asked to think about that in this meeting, and I have been thinking hard.

I find myself thinking about it in terms that might seem quite unrelated. I have become interested in food studies, and attended some meetings of late. At one, I found myself listening to a panel of artisanal food makers, people who were treating the production of food as something of deep cultural and social worth. They were a bit unclear about what specifically they were valuing: the individualized small production? Not necessarily. The classic, traditional techniques? Not always. As I listened to them try to decide what was the "essence" of artisanal food production, what made it meaningful, I was reminded of the ways that midwives talk about birth. Some work in big impersonal hospitals, and yet still claim to be practicing something that should be called midwifery. Some use newer technologies and interventions. Some work in groups and find themselves attending women they never met before. What is it that they are clinging to that marks the essence, the essential truth of midwifery?

And that is the question I hear at this conference, and among the breastfeeding community in general – lactation consultants, La Leche League people – what are we clinging to? If we lose breastfeeding, they are saying, something precious will be lost. This is, I do believe, not all about rationality, though we cling to that, as do the artisanal food makers and the midwives. We can make good strong arguments from the perspective of health, of outcome, of good scientific data. And yet, if they perfected commercial industrial food products, if they perfected cesarean sections, if they perfected artificial breast milk supplements – if all of these were made just as safe and healthy as the "natural" alternatives – I would be left without some of the good arguments I use, but I would not be satisfied. We can make claims to health and to choice: but they are rationalizations we are using because – at root, fundamentally – we believe that the woman in that room with the baby at her breast represents something precious and valuable. We need to make what arguments we can; we need to draw on whatever works in our society; but I think we also need to be honest with ourselves. We need to think hard about where are we coming from and what we are hanging on to, what we can use but not be *used by *as we move ahead, as we try to make that room a safe space.

## Competing interests

The author declares that she has no competing interests.
